# Allied health growth: what we do not measure we cannot manage

**DOI:** 10.1186/s12960-015-0027-1

**Published:** 2015-05-14

**Authors:** Daniela Solomon, Nicholas Graves, Judith Catherwood

**Affiliations:** Institute of Health Biomedical Innovation (IHBI), School of Public Health and Social Work, Queensland University of Technology, 60 Musk Ave. Kelvin Grove, Brisbane, 4059 Australia; Royal Brisbane and Women’s Hospital, Dr. James Mayne Building, Bowen Bridge Rd., Herston, QLD 4029 Australia

**Keywords:** Allied health, Registration, Service provision

## Abstract

**Background:**

Data describing the Australian allied health workforce is inadequate and so insufficient for workforce planning. National health policy reform requires that health-care models take into account future workforce requirements, the distribution and work contexts of existing practitioners, training needs, workforce roles and scope of practice. Good information on this workforce is essential for managing services as demands increase, accountability of practitioners, measurement of outcomes and benchmarking against other jurisdictions. A comprehensive data set is essential to underpin policy and planning to meet future health workforce needs.

**Discussion:**

Some data on allied health professions is managed by the Australian Health Practitioner Regulation Agency; however, there is limited information regarding several core allied health professions. A global registration and accreditation scheme recognizing all allied health professions might provide safeguards and credibility for professionals and their clients.

**Summary:**

Arguments are presented about inconsistencies and voids in the available information about allied health services. Remedying these information deficits is essential to underpin policy and planning for future health workforce needs. We make the case for a comprehensive national data set based on a broad and inclusive sampling process across the allied health population.

## Background

The National Registration and Accreditation Scheme (NRAS) for Health Professionals was introduced in 2010. Ten professions previously registered in every state and territory joined the scheme upon commencement, and four subsequently, with representative national boards overseen by The Australian Health Practitioners Regulation Agency (AHPRA) [1]. Consequently, many established allied health professions have been excluded from the scheme: dietetics, speech pathology, audiology, exercise physiology, orthotists/prosthetists, social work and sonography. This is despite the Productivity Commission recommending at the outset of consultations that registration should occur at as broad a level as possible, consistent with maintaining quality and safety [2]. Over 50 000 practitioners remain outside of the framework of provider accountability and hence patient safety [3]. In February 2011, the Australian Health Ministers Advisory Council (AHMAC) released a consultation paper recommending deferring consideration of inclusion of any additional professions [4]; some 20 proposals from health professions seeking inclusion in the NRAS have been adjourned, despite having addressed the 6 benchmarking criteria underscoring the public benefit of improved practitioner regulation as set out by the Intergovernmental Agreement for the NRAS [5]. Thus, less than a quarter of 50+ health professions as defined by the Health Professionals and Support Services Award 2010 are represented [3].

## Main text

### Current registration

Public trust is predicated on the expectation that a formal regulation structure exists which provides recognition of qualifications, minimum entry standards, assurance of practice standards, a code of conduct and ethics and an avenue for complaints as argued by the National Alliance of Self-Regulating Health Professions (NASRHP) [3]. A recent survey demonstrated that the general public believe they have far greater protection against inappropriate and poor practice with respect to allied health practitioners than they do [6]. The more than 6 million visits to AHPRA’s website and its online register of practitioners in addition to the 310 000 customer service phone calls in the first half of 2011 suggest a requirement for a minimum level of transparency. The role of AHPRA as public safeguard has been emphasized in an analysis of post-registration data by Lin and Gillick [7].

Professional indemnity insurance is not mandatory for several non-AHPRA allied health professions (Table 1). Representative bodies for these professions provide an important regulatory role; however, association membership varies from state to state and between disciplines, with estimates of 60% for speech pathologists to 80% of dieticians [8]. Accreditation programmes are entirely voluntary. For self- or unregulated professions, few of the components of professional accountability are mandatory or enforceable [8]. Where clinical practice guidelines exist, adherence to these professionally defined standards is inconsistent [8]. There is no legislated registration for disciplines such as social work even though there are an estimated 22 000 [9] social workers employed in a diverse range of settings, with some of the most disadvantaged and vulnerable members of the community utilizing their services. Social work remains the largest unregistered health profession, providing services often in rural and remote locations where they are the sole provider of essential counselling and therapy to communities increasingly burdened by mental health issues, yet less than 40% of social workers belong to their professional association at a time when social workers are playing a critical role in the management of mental health disorders [6]. Although a self-regulatory system currently exists within the Australian Association of Social Work (AASW) for managing ethics complaints, only members can be investigated through this process, with the most severe penalty provided under this system being exclusion from eligibility for membership, enabling practise as a counsellor or therapist. The problems associated with loopholes whereby practitioners switching professional titles can evade the regulatory net have been well-documented [7]. Published statistics for the widely accessed Medicare-subsidized Enhanced Primary Care and Chronic Disease Management (CDM) schemes show dieticians among the highest allied health service providers for each year [10], yet dieticians have no requirements to be registered in any state or territory and, thus, no legally enforceable set of probity, qualification and practice standards and no measure of certainty of competence and ethical practice for the members of the public. Registration of speech pathologists in Queensland was in fact recently discontinued by the only state government formerly requiring it, despite the growing need for speech pathology intervention [11].

**Table 1:** **Allied health professions (non-AHPRA) membership and coverage**

	**Audiology**	**Dietetics**	**Orthotics/prosthetics**	**Speech pathology**	**Sonography**	**Social work**
Current publicly accessible national register of registered practitioners	List of clinics and public and private services by region	Yes, including expelled/suspended members	Yes	Yes	No	Yes, including list of conditional and ineligible members (ethics breach)
Practitioners represented (approx.%)	98%	80%	75%	60%	70%	40%
Accreditation status available	No	Yes	Yes	Yes	No	Yes
Professional indemnity	Mandatory for practitioners in private practice and contract or sessional work or if employed by DVA, WorkCover, etc.	Not mandatory	Not mandatory PI, PL and products liability recommended by AOPA	Not mandatory	Not mandatory. PI and PL provided to ASA members only	Not mandatory. PI and PL provided to AASW members only
Complaints^a^				Referred to senior advisor professional issues	Referred to disciplinary committee	Ethics Complaints Management Process

AASW, Australian Association of Social Workers; DVA, Department of Veteran Affairs; PI, Private Insurance; PL, Public Liability; AOPA, Australian Orthotic Prosthetic Association; ASA, Australian Sonographers Association.

^a^Only applies to members of professional organizations. Complaints about non-members directed to the relevant state government service.

### Workforce data—getting ahead of the curve

There is currently no comprehensive national source of allied health workforce data. This has implications for current and future health workforce planning and policy development for the health sector, as robust, reliable and timely data are essential to successful health workforce planning [12]. This has been acknowledged in the US where minimum data sets are being developed at a national level through the Health Resources and Services Administration National Centre for Health Workforce Analysis [13], and in England, the Department of Health has the Centre for Workforce Intelligence [14] as the national authority on workforce planning. The recent ‘Review of Australian Government Health Workforce Programs, Mason Review’ available from the Australian Department of Health Website dealt with Australian Government Health Workforce Programmes and made the point that reliable data sources are limited for the allied health workforce. This review called for better data collection across settings to provide information for policy development and made special mention of the disability sector that is now being reformed by the introduction of the National Disability Insurance Scheme.

Workforce Reports (National Health Labour Force Series) with a range of data have been produced by the Australian Institute of Health and Welfare (AIHW) [15] for a limited number of allied health professions (physiotherapists, occupational therapists and podiatrists), but no such reports have been produced for audiology, dietetics, speech pathology or radiography, although this is changing with the introduction of the HW 2025 series by Health Workforce Australia which will provide coverage of 40 professions [16]. Accurate reporting of current service provision and determining projections regarding future supply and demand is not possible for a number of allied health professions. For example, there is currently no substantive data available across the professions regarding hours worked with clients in the format of caseload information regarding number of equivalent full time (FTE) positions per client age and/or diagnostic category or FTE per clinical setting [11]. Some attempts to establish benchmarks in staffing levels in specific health areas such as palliative care have been made, but few examples of published staffing ratios exist [17], with figures based on therapist-to-bed or therapist-to-patient ratios rather than evaluation of adequacy of current service provision and projections beyond this [11]. This highlights the necessity for benchmarking in terms of the allied health workforce in general, particularly with regard to service demand and prevalence of clinical population types such as neurological and rehabilitation patients, especially given the projected growth in illnesses such as dementia. Australian Labour Force Survey data [18] indicate that the number of ‘other allied health workers’ (dieticians, occupational therapists, podiatrists, speech professionals, audiologists, orthoptists) has increased in terms of FTE number (based on a standard working week of 35 hours) but decreased in terms of FTE rate (FTE per 100 000 population), with consequent workplace shortages failing to meet the identified need and maldistribution of service provision to rural, remote and indigenous communities. From 2001 to 2006, service provision in nuclear medicine grew by 11.8%, but the workforce size decreased by 4.9% [19]. This is also reflected in international data; successive reports by the General Dental Council (UK) showed that, while the dental technician workforce was declining, workloads were increasing [20,21] along with dissatisfaction due to increased workload pressures [22]. In many cases, data on gender are not recorded by state and territory boards; this is being addressed for AHPRA members but has implications for a workforce that is highly feminized. Changes in the delivery settings away from institutional-based and outpatient care to community settings ar also a driving factor in allied health affecting employment in countries such as the US and Australia [23,24].

### Stayers, leavers and returners—supporting a flexible and responsive workforce

Developing a comprehensive national data set requires a broad and inclusive sampling process across the allied health population. Although professional associations outside the AHPRA ambit collect data about their members, association membership is not mandatory, and as such, this data is also incomplete. Surveys conducted to investigate demographics, employment, education and factors affecting recruitment and retention of allied health professionals have had low-response rates both here and internationally [22,25,26]; the recently published South Australian Allied Health Workforce (SAAHW) survey had an estimated combined response rate of only 18.3%, with occupational response rates ranging from 57% for dietetics to 1.3% for audiology [27]. Some professions provided no responses at all. Evidence concerning recruitment, retention and turnover in the allied health literature is sparse. Amid concerns of an ageing workforce in several occupations, the 2011 census shows proportionately higher numbers of allied health professionals in the younger age groups (up to 39 years). But put in context, these figures are less reassuring—research has shown the impact of generational issues in a tight labour market and that attrition and turnover are higher among Generation Y employees [19,28]. A recent allied health survey indicated that, although of the 42% who intended leaving their current job within 5 years 28% gave retirement as the reason [27], a significantly larger proportion of the Generation Y respondents indicated that they intended leaving their job within 2 years (46%). Surveys have shown that almost half the nuclear medicine technologist workforce is aged less than 30 years; many leave before completing 10 years in practice [19]. An earlier study into the same profession found a third of respondents to that survey had less than 3 years experience, creating a constantly young workforce with limited experience [29]. Similarly, published data on speech pathology indicated that those aged under 34 years were more likely to be looking for a new career and that 31% of those surveyed intended to change jobs [30]. No planning has been done at a national level with respect to this generational point at issue [31].

### Professional development and service delivery

It is generally accepted that Australia will continue to experience increasing demand for health-care workers and at a rate that will challenge Australia’s training and service delivery systems [32]. Student registration was launched nationally for the first time in Australia in 2011, but again, this only applies to professions registered under the NRAS agreement. Although the supply of graduates from universities is gradually increasing, government policy and benchmarks remain unchanged [33]. Profession accreditation requirements are one of the key issues affecting universities in virtually all health profession courses; a major determining factor in setting clinical placement requirements is professional accreditation [34]. This increase in university places has not necessarily been paralleled by clinical placements or an increase in supervisor numbers, although once again this is being addressed by the Health Workforce Australia clinical placement data set [35]. Access to clinical placements is seen as a constraint on growth in all jurisdictions. This can be seen in professions such as podiatry. At 3 738 members, it is by far the smallest of the core allied health group, with one of the fewest numbers of students in approved programmes of study [36]. Statistics from Medicare Australia show that demand for podiatry services increased more than five times over the 2008–2009 period in some areas. Additionally, national labour force statistics show podiatry has a growth outlook of 302% over 2 years [37]. The factors driving workforce shortages compound one another—university planning decisions are influenced primarily by student and then workforce demand, which itself is influenced by perceptions of likely employment outcomes, and population and demographic change [34]. However, the current training system has insufficient capacity and structure to provide workers in a timely manner to meet demand, further driving worker shortages. US research has highlighted the need for greater information on ‘career ladder programmes’ to make the concept of career progression a reality for entry-level and low-wage health-care workers [23].

### Benefits of registration

Beyond the need for current and consistent data to use in workforce planning, there are additional advantages to increased coverage of registration for allied health, in particular, the need to establish clear and unambiguous standards of professional competence. A registration and accreditation scheme as provided by AHPRA across all allied health professions provides the necessary safeguards for professionals and their clients to ensure quality services are provided by people with the requisite skills and knowledge to practise, as has been established in the UK with the Professional Standards Authority for Health and Social Care [38]. It provides standardization, credibility and greater professional identity [39]. This increases public confidence that variations in quality of care are minimized and safety is optimized [38]. The benefit and value to society is self-evident. Educational programmes benefit from an individual credentialing entity to prepare students for certification [39].

Research both here and overseas has demonstrated that allied health professionals are more likely to stay with an organization when they are able to attain higher levels of competency and professional development, there is good peer support in the workplace and inter-professional collaboration and workplaces encourage ‘continuous learning cultures’ [28,40]. UK professional bodies require higher education institutions to provide evidence of inter-professional education as a key process to enable collaboration [41], which is recognized as benefiting both the professional experience and patient care [42]. Although the number of allied health professionals with higher degrees is growing, research funding is still extremely small when compared to that in other areas of health care [43]. A requirement of the move towards an evidence-based culture is a critical mass of health-care professionals either in a position to conduct research or to implement scientific findings [44]. Without registration and the emphasis on evidence-based practice programmes [45], associated allied health practitioners may find themselves left behind due to lack of research skills, research not transferable to practise, and lack of managerial support for ongoing research. Smaller professions with less visibility are also at risk of reduced funding for workforce development and education [46].

### Costs of registration

There are likely to be significant costs to a national registration scheme. Apart from the bureaucracy required to manage the data collection and good governance, there will be costs imposed on allied health professionals required to fill in forms, gather data and participate. Acting on the complaints and issues that arise will also engender resources and could open up a range of high costs arising from litigation. Privacy concerns and confidentiality issues are likely to make registration and accreditation yet more complex and cumbersome.

### International relevance

The extent to which data are available for other countries is not surprisingly highly variable. A summary can be accessed from the OECD website [47]. At one extreme are countries such as Poland and Sweden who have no information to describe dentists, pharmacists, physiotherapists, psychologists, dieticians, audiologists and speech therapists and laboratory assistants. This clearly is inadequate for workforce planning. In contrast, Chile has the National Health Human Resources Information System of the public sector that includes all public hospitals, but no data are available for the private health sector. The Czech Republic benefits from annual reports produced by the Institute of Health Information and Statistics of the Czech Republic. Information arises for individual hospitals and other therapeutic institutes by profession, and the measurements of staff are made by head counts and full-time equivalents to discriminate between employees on payroll and contractual workers. Denmark reports head counts via the National Board of Health and only public sector staff are included. Full descriptions of data sources are available from the OECD for Austria, Belgium, Canada, Germany, Hungary, Iceland, Italy, Luxembourg, Mexico, Netherlands, New Zealand, Switzerland, Turkey, United Kingdom and the United States. The relevance of this work done for the Australian setting to other countries will depend on the completeness and quality of the information collected in each setting.

## Conclusions

Health workforce shortages in Australia will produce unwanted public health consequences if not addressed by policy makers [31], including contraction and even disappearance of health services in areas already experiencing major problems of access, such as remote and rural regions and the more socially and economically marginalized suburbs in urban centres [48].

Development of an efficient and effective coordinated, multidisciplinary health-care system in Australia requires a combination of evidence-based strategies and reliable workforce data to ensure that the health professions can work together in the interest of improved patient outcomes. Currently, robust evidence about the allied health professions is not available to support this objective. A wider range of professions might be included under AHPRA and representative bodies strengthened to improve the level of registration and accountability of health professionals. Worth debating in Australia is the establishment of a minimum data set for allied health professionals. Lessons could be learned from other jurisdictions such as the US Center for Health Workforce Analysis and the English Centre for Workforce Intelligence.
